# Gut-placenta axis: gut microbiota metabolites may play a role in regulating maternal-fetal immune tolerance and the pathogenesis of adverse pregnancy outcomes

**DOI:** 10.3389/fimmu.2026.1960306

**Published:** 2026-09-11

**Authors:** Ruxue Zhou, Shenglan Wang

**Affiliations:** 1 Obstetrics and Gynecology, Clinical Medical College of Qinghai University, Xining, Qinghai, China; 2 Obstetrics, Qinghai Red Cross Hospital, Xining, Qinghai, China

**Keywords:** adverse pregnancy outcomes, gut microbiota, gut-placental axis, maternal-fetal immune tolerance, microbial metabolites, short-chain fatty acids, trophoblasts

## Abstract

Pregnancy is a unique physiological stage characterized by the coordinated remodeling of maternal metabolism, immunity, and endocrine systems. Previous studies have primarily focused on the local uterine microenvironment, neglecting the role of the maternal gut microbiota in mediating distal, cross-organ regulation via the gut-placental axis. The concept of the gut-placental axis was formally proposed in 2018 and has attracted widespread research attention in recent years. Studies using germ-free animals, fecal microbiota transplantation (FMT), and *in vitro* experiments with human multi-omics cohorts suggest that live gut bacteria generally cannot colonize the placenta, and that soluble microbial metabolites mediate bidirectional signaling between the gut and the placenta. Pregnancy-related factors such as a high-sugar, high-fat diet, antibiotic use, and obesity can induce gut microbiota dysbiosis and intestinal barrier dysfunction, leading to decreased levels of potentially protective metabolites such as short-chain fatty acids (SCFAs) and tryptophan derivatives, while pro-inflammatory molecules such as lipopolysaccharide (LPS), and trimethylamine N-oxide (TMAO) to accumulate in the bloodstream. These substances cross the placental barrier, disrupting maternal-fetal immune homeostasis, inhibiting trophoblast function and spiral artery remodeling, and are statistically associated with adverse pregnancy outcomes such as recurrent spontaneous abortion (RSA), preeclampsia (PE), gestational diabetes mellitus (GDM), fetal growth restriction (FGR), and spontaneous preterm birth. This article is a narrative review that systematically elucidates the - placental axis for substance transport and the potential molecular mechanisms by which metabolites regulate maternal-fetal immune balance; it maps out the pathological pathways through which microbiota dysbiosis mediates various adverse pregnancy outcomes; it objectively analyzes controversies and gaps in areas such as placental microbial colonization, bidirectional feedback loops, and evidence for clinical interventions; and it proposes a future research framework centered on multi-omics candidate biomarkers and stratified microbiome interventions. Microbial metabolites are promising candidates for non-invasive biomarkers; stratified microbiome interventions are merely potential prevention and control strategies suggested by laboratory and small-scale clinical studies. There is still a lack of unified, standardized clinical protocols, and large-scale, long-term, randomized controlled trials (RCTs) are needed to validate their safety and efficacy, thereby providing a theoretical foundation for subsequent mechanistic and translational research on maternal-fetal microbiome interactions.

## 1 Introduction

A normal pregnancy is essentially a process of semi-allogeneic transplant tolerance: after an embryo carrying paternal antigens implants, the maternal immune system must moderately suppress the cytotoxic immune response and establish a bidirectional immune balance that fluctuates dynamically throughout pregnancy, balancing tolerance to the allogeneic embryo with the mother’s defense against infection. This is not a state of continuous, simple immune suppression; rather, it preserves the mother’s baseline ability to fight infection. This steady state is known as maternal-fetal immune tolerance (1). Traditional research on reproductive immunology has largely been limited to the local interactions between the uterine microbiota, decidual immune cells, and placental trophoblasts, while the potential systemic, long-distance effects of the maternal distal gut microbiota on the maternal-fetal interface homeostasis have long been overlooked (2). Since 2018, the -placental axis was proposed, this field has attracted widespread research attention, challenging the long-held belief that the gut and placenta are independent organs. Numerous studies using germ-free animals, isotope tracing, and human cohort studies suggest that the maternal gut microbiota does not require live bacteria to colonize the placenta across the barrier; instead, it may achieve bidirectional signaling between the gut and placenta solely through small-molecule soluble metabolites (3); Animal models and *in vitro* experiments suggest that microbial metabolites may be involved in regulating maternal-fetal immune homeostasis; however, causal evidence in humans for most of these pathways remains insufficient, and the existing complete mechanisms of action are largely based on integrated inferential hypotheses.

Multiple internal and external factors—such as hormonal fluctuations during pregnancy, a high-sugar, high-fat diet, exposure to broad-spectrum antibiotics, chronic psychological stress, and pre-pregnancy obesity—may disrupt the stability of the gut microbiota (4): Species in the phylum Firmicutes that produce butyric acid—such as Faecalibacterium prausnitzii and Clostridium butyricum—have protective effects; Excessive proliferation of certain Gram-negative opportunistic pathogens within the Proteobacteria phylum (such as Proteus mirabilis and Escherichia coli) can release LPS, thereby inducing inflammation; functional differences among genera and species within the same phylum are extremely significant, so the phylum as a whole cannot be used to make generalizations; downregulation of intestinal tight junction proteins (occludin, claudin) leads to a pathological state characterized by increased intestinal permeability (5). Microbiota dysbiosis simultaneously triggers two types of cascading changes: First, concentrations of potentially protective metabolites—such as SCFAs, tryptophan derivatives, and secondary bile acids—decrease; second, pro-inflammatory molecules such as LPS, TMAO, and toxic bile acids enter the bloodstream in large quantities and, via the systemic circulation and the placental transport system, reach the maternal-fetal interface, where they may disrupt the immune balance of the decidua and potentially inhibit normal placental development (6, 7).

Preeclampsia is a common pregnancy complication worldwide, with a prevalence of approximately 4.43% (95% CI: 3.73%–5.20%) among pregnant women; however, there are significant variations across regions and diagnostic criteria. This condition significantly increases the risk of adverse maternal and perinatal outcomes (8), Tomkiewicz et al. (2023) noted in a European RSA cohort that approximately 50% of patients still had no clear cause identified after systematic screening (9); this proportion varies depending on the scope of screening tests and the ethnic composition of the population. Complications such as fetal growth restriction (FGR), preterm birth, and gestational diabetes (GDM) cause a large number of short-term maternal and fetal injuries each year, as well as long-term risks of metabolic and immune disorders; existing local placental mechanisms cannot fully explain the etiology of all cases (10). The gut-placenta axis contributes to the pathogenesis of pregnancy complications through the gut microbiome in the distal intestine. Microbial metabolites offer the advantages of noninvasive detection and targeted intervention, opening up a new direction for screening for adverse early pregnancy risks and for precise prevention and treatment. However, this approach requires validation through large-scale randomized controlled trials (RCTs), its long-term safety remains unclear, and it is not currently recommended for routine clinical use (2).

Existing reviews on the gut-placental axis published between 2018 and 2026 have significant shortcomings: most studies only discuss the unidirectional pathogenic mechanisms by which gut microbiota dysbiosis causes placental damage, and very few systematically dissect the gut - placental bidirectional pathological positive feedback loop; there is also a lack of a standardized evidence-grading system for the pathways of various microbial metabolites, with no consistent distinction made between causal evidence from animal studies, population-based association evidence, *in vitro* cellular hypotheses, and integrative inferences; furthermore, most studies focus solely on the pathogenic phenotypes resulting from microbial dysbiosis, lacking comparative analyses that simultaneously examine the physiological adaptive homeostasis of the gut microbiota during normal pregnancy.

Compared with similar reviews published in recent years, this review has the following three distinctive features: First, it innovatively integrates multidimensional research data from germ-free animals, FMT, human multi-omics cohorts, and *in vitro* placental organoid models, and introduces a unified four-tier evidence grading system to clearly distinguish empirical conclusions from theoretical hypotheses; Second, it simultaneously establishes two parallel lines of argument—one on “physiological adaptation of the gut microbiota during normal pregnancy” and the other on “pathological damage caused by microbiota dysbiosis”—to comprehensively compare the distinct signaling patterns of the gut-placental axis under physiological homeostasis and pathological imbalance; Third, it thoroughly dissects the bidirectional regulatory loop comprising positive gut-to-placental signaling and negative placenta-to-gut feedback, objectively noting that the feedback loop whereby the placenta regulates the intestine is derived solely from the integration of multiple studies and lacks direct *in vivo* tracing evidence; clearly distinguishing established pathways from hypotheses awaiting verification; and constructing a complete logical chain spanning basic molecular mechanisms, disease pathological pathways, areas of controversy and gaps in the field, to directions for stratified microbiome intervention. This approach addresses the shortcomings of existing reviews, which are characterized by one-sided arguments, ambiguous levels of evidence, and a lack of physiological homeostasis controls.

This paper sets two clear objectives: ① To systematically review the bidirectional signaling pathways of the gut-placental axis and, based on different experimental systems—including germ-free animals, fecal microbiota transplantation (FMT), human cohorts, and placental organoids—to categorize, by tier, the standardized levels of evidence suggesting that metabolites may be involved in regulating maternal-fetal tolerance; ② To objectively analyze long-standing academic controversies in the field and identify key gaps in evidence at each stage, while proposing only a framework for future research exploring tiered microbiome interventions—without establishing or recommending mature diagnostic and treatment protocols ready for direct implementation in routine clinical practice. This narrative review systematically elucidates the substance transport pathways of the gut-placental axis, the molecular mechanisms by which metabolites regulate maternal-fetal tolerance, and the pathophysiological mechanisms by which microbiota dysbiosis mediates various adverse pregnancy outcomes, while objectively outlining existing limitations and challenges in clinical translation within the field.

## 2 Research methods

### 2.1 Definition of review types

This paper is a narrative review and does not follow the standardized procedures of a systematic review, such as a predefined comprehensive search strategy, comprehensive and undifferentiated literature screening, quantitative meta-analysis, or risk of bias assessment. With the gut-placental axis as its central theme, it integrates representative basic experimental and clinical observational studies in the field, focusing on elucidating mechanisms, reviewing controversies in the field, and outlining future directions for translation. It does not conduct quantitative meta-analyses.

### 2.2 Guidelines for literature search

Databases searched: PubMed, Web of Science, Embase, Cochrane;

Search time frame: 2018 (when the concept of the gut-placenta axis was formally proposed) through July 2026;

Search Terms: Gut-Placenta Axis, Intestinal Microbiota, Microbial Metabolites, Short-chain Fatty Acids, Maternal-Fetal Immune Tolerance, Preeclampsia, Recurrent Spontaneous Abortion, Gestational Diabetes Mellitus, Fetal Growth Restriction, Preterm Birth, Placental Organoid, Fecal Microbiota Transplantation;

Inclusion Criteria: ① Functional validation studies in germ-free animals and fecal microbiota transplantation studies related to the gut-placenta axis; ② Human multi-omics, case-control, and prospective pregnancy cohort studies; ③ *In vitro* molecular experiments on placental organoids and trophoblasts; ④ Randomized controlled intervention trials on dietary fiber, probiotics, and postbiotics during pregnancy; Priority will be given to high-impact original mechanism-based literature published within the past 5 years; studies that are purely cross-sectional descriptions of the microbiome without any mechanistic analysis will be excluded.

Exclusion criteria: ① Single-case reports and small-sample case studies; ② Duplicate publications and highly overlapping papers from the same research team; ③ Cross-sectional studies that merely describe differences in microbial abundance without exploring underlying mechanisms; ④ Preprints on medRxiv and bioRxiv (retained but labeled as “not peer-reviewed”).

### 2.3 Criteria for classifying evidence

All inferences regarding biological mechanisms throughout this document are labeled with the corresponding evidence level, which is categorized into 4 levels:

Level 1 (Evidence of Causality): Sterile animal models, functional animal studies on fecal microbiota transplantation (FMT), and randomized controlled clinical trials in humans; causal effects can be established within the respective experimental systems, but results from rodent studies cannot be directly extrapolated to humans (11).

Level 2 (Associative Evidence): Human case-control studies, nested/prospective cohort studies, and plasma/fecal multi-omics association analyses; these can only demonstrate statistical associations, cannot fully eliminate confounding factors, and cannot establish causality (12–14).

Level 3 (*In vitro* hypothetical evidence): *In vitro* cellular experiments using placental organoids, trophoblast cells, and decidual immune cells; these experiments only validate the effects of a single molecular pathway in an *in vitro* setting, and their role under physiological *in vivo* conditions remains uncertain (15).

Level 4 (Integrated Inference Hypothesis): A pathway model derived by synthesizing findings from multiple independent animal and human studies, lacking direct validation through *in vivo* tracing (e.g., the feedback loop in which placental extracellular vesicles negatively regulate the gut microbiota); this is merely a theoretical hypothesis (16).

### 2.4 Principles for literature synthesis

Studies addressing both the physiological adaptation of the microbiota during normal pregnancy and the pathogenicity resulting from microbiota dysbiosis were included in a balanced manner; rather than merely listing linear pathogenic pathways, the review simultaneously addressed conflicting and controversial experimental findings within the field; studies with insufficient sample sizes or low levels of evidence were discussed only as supplementary information and were not used to support the derivation of core mechanisms.

## 3 The intestine-placenta axis: a bidirectional pathway for substance exchange

During a physiological pregnancy, estrogen and progesterone gently remodel the maternal gut microbiota, enriching beneficial bacteria that produce short-chain fatty acids (SCFAs)—such as Bifidobacterium and Akkermansia mucinicola—thereby elevating baseline levels of protective metabolites in the circulation and helping to maintain baseline immune tolerance between mother and fetus; Only when external factors (high-sugar, high-fat diets, antibiotics, obesity) disrupt this physiological homeostasis do they induce dysbiosis, increased intestinal permeability, and adverse pregnancy outcomes.

**Figure 1:**
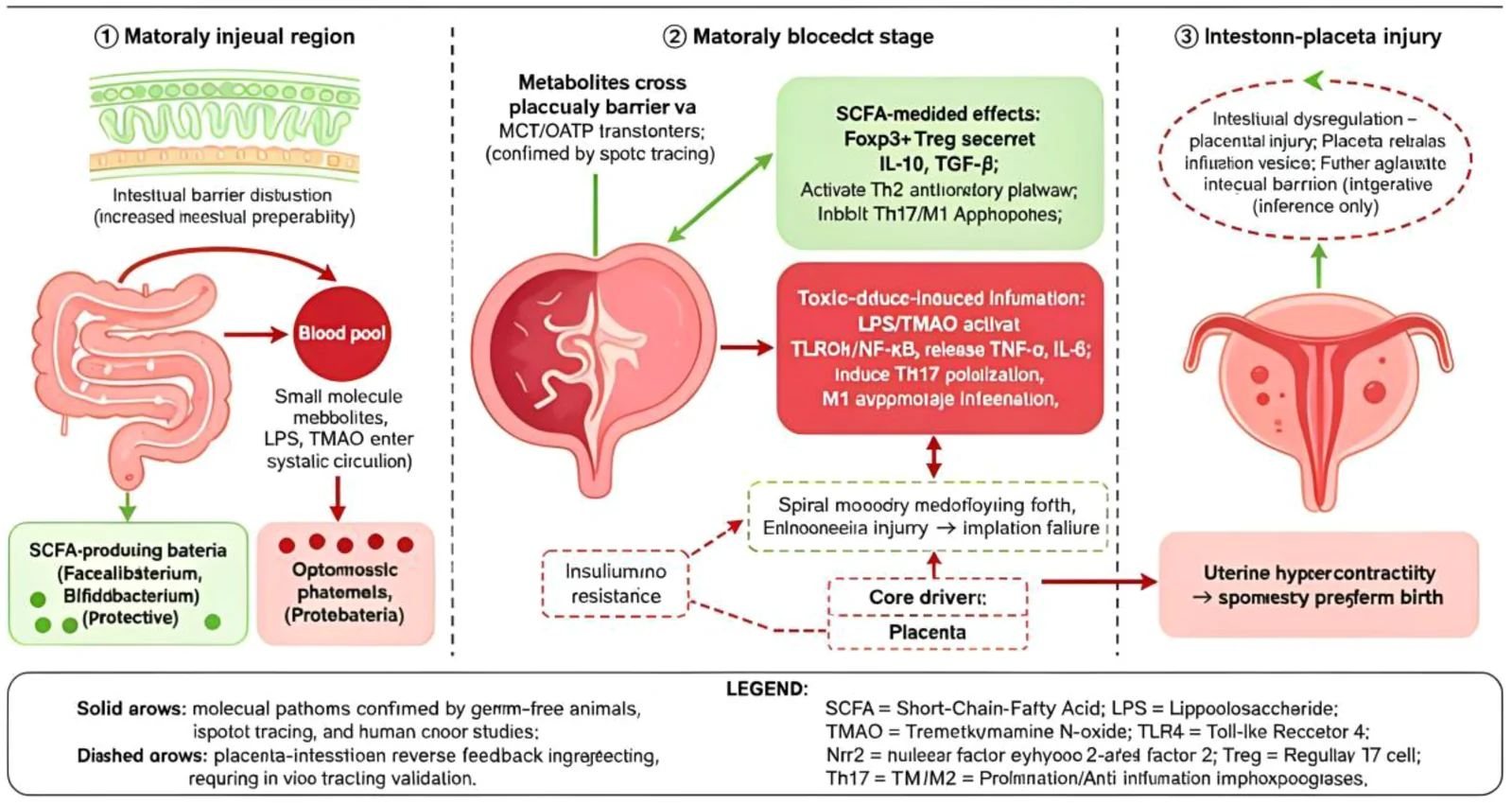
Intestinal barrier dysfunction mediates inflammatory pathways at the gut–placenta interface.

Current mainstream academic consensus: There is no reliable evidence to support the large-scale colonization of the placental tissue by live gut bacteria; under physiological conditions, the placenta maintains a sterile microenvironment, and the trace amounts of bacterial nucleic acids detected in placental sequencing are primarily attributable to contamination from the birth canal during delivery and background interference from sequencing reagents (17). Communication along the gut-placenta axis primarily relies on the long-distance transmission of soluble microbial metabolites and bacterial extracellular vesicles via body fluids (18); In cases of severe increased intestinal permeability, bacterial fragments and cellular components may transiently translocate to the maternal-fetal interface; however, they cannot form a stable placental microbiome. The complete signaling pathway comprises three layers: the intestinal metabolic synthesis layer, the systemic circulation transport layer, and the maternal-fetal interface response layer. Concurrently, there is retrograde signaling from the placenta to the intestine, forming a bidirectional regulatory loop that provides a new theoretical perspective for understanding distal immune regulation at the maternal-fetal interface (1). Abnormalities in the gut-placental axis regulatory network observed in preeclampsia also support this perspective from a pathological standpoint (5) as shown in **Figure 1**.

### 3.1 Intestinal metabolic synthesis and intestinal barrier filtration

Substrates fermented by the gut microbiota produce large quantities of small-molecule bioactive metabolites, which, via the gut-placental axis, regulate the function of placental trophoblasts and the maternal-fetal immune balance (19); the intact intestinal epithelium serves as a selective barrier, preventing intestinal microorganisms and macromolecular antigens from crossing the intestinal wall, while allowing only small-molecule nutrients and microbial metabolites to be absorbed into the bloodstream (20). Microbial dysbiosis can damage the intestinal mucosal barrier, leading to increased intestinal permeability, which facilitates the systemic translocation of microbial metabolites (21). This continuously activates the Toll-like receptor 4 (TLR4)/nuclear factor κB (NF-κB) inflammatory pathway, amplifying inflammatory damage to the placenta, and may be one of the potential upstream triggers of various pregnancy complications (22).

### 3.2 Cross-barrier transport from the circulatory system to the placenta

Trophoblast cell membranes in the placenta specifically express transporters and receptors that mediate the passage of microbial metabolites across the placental barrier: the monocarboxylate transporters (MCT) MCT1 and MCT4 are responsible for the transmembrane transport of short-chain fatty acids (SCFAs)—acetate, propionate, and butyrate; G-protein-coupled receptors 41/43 (GPR41/GPR43) are SCFA-specific membrane receptors that are highly expressed in trophoblasts and decidual macrophages and are involved in the pathophysiological process of preeclampsia (23); Organic anion transporting polypeptides (OATPs), primarily organic anion transporting polypeptide 2B1/3A1 (OATP2B1/OATP3A1), which are highly expressed in the placenta, mediate the transplacental transport of bile acids (24) and tryptophan derivatives (25).

Isotope-tracing experiments have confirmed that labeled butyrate supplemented during pregnancy can be detected in the livers, brain tissues, and placental tissues of fetal mice, directly demonstrating that SCFAs can cross the placental barrier and act simultaneously on both placental tissue and fetal target organs (Level 1, isotope-tracing animal studies, directly verifying transplacental transport of SCFAs) (26). The aforementioned transporters and receptors collectively constitute the molecular pathway for the unidirectional delivery of intestinal metabolites to the maternal-fetal interface. During normal pregnancy, the expression of placental transporters is upregulated, ensuring a continuous and adequate supply of protective metabolites to the maternal-fetal interface; following gut microbiota dysbiosis, the expression of transporters in the MCT and OATP families is downregulated, resulting in a significant decrease in the efficiency of placental delivery of SCFAs and indole derivatives, while passive diffusion of toxic molecules increases.

It should be noted that the effects of gut microbiota metabolites on the maternal-fetal interface can be divided into two relatively independent yet synergistic pathways: the first is a direct transplacental pathway, in which SCFAs, tryptophan derivatives, bile acids, and other metabolites are actively transported across the barrier via transporters such as MCT and OATP, directly binding to GPR43, AhR, and TLR4 on the surfaces of trophoblasts and decidual immune cells to regulate intracellular signaling. This has been directly validated by isotope-tracing animal studies (Level 1) and is further supported by *in vitro* organoid experiments (Level 3);The second mechanism is an indirect pathway mediated by systemic inflammation and metabolic disorders in the mother, Once LPS, TMAO, BCAAs, and other substances enter the bloodstream, they first induce systemic low-grade inflammation, vascular endothelial dysfunction, and insulin resistance in the mother; they then indirectly affect the placenta via uterine spiral artery perfusion and the decidual microenvironment. This pathway is primarily supported by population-based observational evidence (Level 2); however, the causal chain is lengthy, and confounding factors are difficult to fully control. These two pathways are not mutually exclusive, but as their levels of evidence and limitations on generalizability differ, they must be evaluated separately and should not be conflated.

### 3.3 The placenta sends feedback signals to the gut (bidirectional axis)

The retrograde signaling from the placenta to the gut forms the theoretical framework for bidirectional regulation of the gut-placental axis; however, there is a significant difference in the completeness of evidence between the two pathways: the pathway by which gut microbiota metabolites act on the placenta via the circulation is supported by multiple direct lines of evidence, including isotope tracing, animal FMT studies, and human plasma metabolomics (Level 1: germ-free animal and FMT animal studies; Level 2: human cohort and population-based association evidence); in contrast, the feedback loop in which inflammatory mediators and extracellular vesicles released by the placenta may participate in regulating the intestinal barrier and microbiota homeostasis is based solely on inferences derived from the integration of multiple independent studies. There is currently no direct evidence from *in vivo* tracing of placental vesicles targeting the intestine; this remains a hypothesis awaiting verification, and significant gaps still exist in the relevant molecular signaling pathways.

The gut-placental axis is not a unidirectional regulatory pathway from the gut to the placenta; a damaged placenta can release extracellular vesicles containing soluble vascular endothelial growth factor receptor 1 (soluble fms-like tyrosine kinase-1, sFlt-1), inflammatory microRNAs, and pro-inflammatory cytokines into the maternal circulation, forming a pathological feedback loop (27, 28). Placental hypoxia can induce the massive release of small extracellular vesicles, which in turn elevates systemic pro-inflammatory factor levels and triggers the hypertensive phenotype during pregnancy. Animal models have validated the regulatory effects of these vesicles on maternal blood pressure and systemic inflammation (Level 1: animal modeling; causal validation limited to the animal level; cannot be directly extrapolated to humans) (29); Placenta-derived extracellular vesicles act at a distance on the intestinal mucosa via the bloodstream, downregulating the abundance of butyrate-producing bacteria, degrading intestinal tight junction proteins, and exacerbating increased intestinal permeability and dysbiosis (30).

Previous cohort studies and animal models have demonstrated that butyrate deficiency in the gut and damage to the intestinal barrier can elevate circulating sFlt-1 levels and induce placental dysfunction; meanwhile, inflammatory signals released by placental damage further exacerbate gut microbiota dysbiosis, ultimately leading to a vicious cycle of positive feedback in which gut dysfunction → placental damage → further gut dysfunction (Level 4, integrated inferential hypothesis). This cascade reaction is merely a theoretical model derived by piecing together findings from multiple independent studies; to date, no single *in vivo* tracing experiment has fully replicated the entire cascade. Therefore, it cannot be used to definitively identify a pathogenic pathway but serves only as a potential hypothesis explaining the progression of preeclampsia and recurrent miscarriage (30, 31). A normally oxygenated placenta releases low-concentration anti-inflammatory extracellular vesicles, which mildly promote intestinal barrier stability; a hypoxic-damaged placenta releases large amounts of pathological vesicles carrying sFlt-1 and pro-inflammatory microRNAs, which downregulate butyrate-producing microbiota and disrupt intestinal tight junctions, creating a positive feedback loop of damage. This complete cascade has not been consistently replicated in *in vivo* experiments and remains a theoretical hypothesis.

## 4 A molecular hypothesis suggesting that gut microbial metabolites may be involved in regulating maternal-fetal immune tolerance

Maternal-fetal immune tolerance homeostasis depends on the balance of three major immune cell types: regulatory T cells (Treg)/the Treg/Th17 ratio, the polarization of type 2 macrophages (M2) versus type 1 pro-inflammatory macrophages (M1), and the normal function of uterine natural killer cells (uNK) (32). Under physiological conditions, various bioactive metabolites produced by the maternal gut microbiota maintain the aforementioned tolerance balance through three parallel pathways: epigenetic modification, activation of membrane receptors, and activation of antioxidant pathways; moreover, there are significant differences in the targeted regulatory pathways of metabolites from different categories of microbiota (33, 34). Following a disruption in the microbial balance, protective metabolites are depleted, toxic molecules accumulate, or the homeostasis of tolerance is disrupted. For each class of metabolites, we simultaneously distinguish between potential physiological protective trends and hypotheses regarding damage caused by pathological imbalances. Please refer to Appendix 1 for details.

### 4.1 Short-chain fatty acids: protective molecules that maintain immune tolerance

SCFAs are produced by Bacteroides, Bifidobacterium, and Clostridium butyricum through the fermentation of dietary fiber; acetate, propionate, and butyrate account for more than 90% of the total. Animal and *in vitro* studies suggest that they possess three functions: anti-inflammatory effects, potential angiogenesis promotion, and fetal epigenetic regulation (33).

#### 4.1.1 Restoring the Treg/Th17 balance in the decidua

Physiological Regulation (Normal Pregnancy): Physiologically adequate levels of butyrate mildly inhibit histone deacetylases (HDACs), moderately upregulate the transcription of forkhead box protein P3 (Foxp3), or induce a moderate number of Tregs to homing to the decidua (35, 36); Tregs secrete interleukin (IL)-10 and transforming growth factor-β (TGF-β), which potentially inhibit Th17 proliferation and IL-17A release, thereby maintaining immune tolerance (35–37). Moderate levels of IL-17 promote the invasion of extravillous trophoblasts (EVTs), indirectly contribute to the remodeling of spiral arteries, and maintain a moderate immune response that neither attacks the embryo nor compromises the body’s ability to fight infection (Level 1, animal studies; Level 3, *in vitro* validation of mechanisms). Pathological Imbalance-Induced Damage Hypothesis: When there is a combined deficiency of SCFAs and tryptophan metabolites, the Th17/Treg balance is disrupted, IL-17-mediated local inflammation is exacerbated, the risk of embryonic loss increases, and this leads to incomplete remodeling of the spiral arteries (33). Hu et al. noted in their review (33) that Brown et al. demonstrated in a germ-free mouse (C57BL/6 background) model that the absence of gut microbiota leads to dysregulation of the IFN-γ/IL-17 response at the maternal-fetal interface, a reduction in RORγt^+^Foxp3^+^ Tregs, and an increased rate of fetal resorption; oral administration of indole-3-methanol (a tryptophan metabolite and AhR agonist) or colonization with Lactobacillus murinus, which produces tryptophan metabolites, restored immune balance at the maternal-fetal interface and reduced the risk of fetal resorption.This study also validated the dysregulation of myeloid-derived suppressor cells (MDSCs), RORγt^+^ Tregs, and tryptophan metabolites in decidual tissue from women with recurrent miscarriage. It should be noted that these data are derived from animal experiments, and values may vary across different mouse strains and human populations (38) (Level 1, germ-free animals; causal validation experiments with repletion interventions).

#### 4.1.2 Regulation of decidual macrophage polarization

Physiological Regulation (Normal Pregnancy): At physiological concentrations, SCFAs inhibit NF-κB-mediated pro-inflammatory signaling via HDAC inhibition, upregulate the antioxidant signaling pathway mediated by nuclear factor erythroid 2-related factor 2 (Nrf2), induce a stable shift in macrophage polarization toward the M2 (tolerant) phenotype, promote the secretion of anti-inflammatory factors, clear apoptotic cell debris, and maintain an inflammation-free microenvironment in the placenta (Grade 2, cohort of healthy pregnant women).

Pathological Imbalance-Induced Damage Hypothesis: When a dysbiotic microbiota leads to a deficiency in short-chain fatty acids (SCFAs), macrophages shift toward the pro-inflammatory M1 phenotype, creating a chronic inflammatory microenvironment in the placenta (39, 40). Massive M1 infiltration is a hallmark pathological feature of preeclampsia, and the large amounts of pro-inflammatory mediators secreted by these cells can disrupt the normal developmental homeostasis of the placenta (39). A clinical cohort study (humans, n = 20 patients with PE vs. n = 20 healthy controls) showed that patients with PE had reduced abundance of gut SCFA-producing bacteria, decreased serum SCFA levels, and upregulated expression of cathepsin C in placental tissue; cathepsin C promotes trophoblast apoptosis and disrupts spiral artery remodeling (40) (Level 2, human case-control cohort, population-based evidence).In an L-NAME-induced preeclampsia rat model, SCFA supplementation can downregulate cathepsin C and alleviate hypertension, proteinuria, and fetal growth restriction (40) (Level 1, animal study). It should be noted that the clinical cohort had a small sample size (n = 20 per group) and measured serum SCFAs rather than fecal SCFAs; values vary across different populations (ethnicity, gestational age, BMI, dietary patterns) and animal strains (rats vs. mice, different PE induction models), so extrapolation should be done with caution (30, 40, 41).

#### 4.1.3 Promoting placental vascular development

Physiological Regulation (Normal Pregnancy): *In vitro* studies have shown that circulating SCFAs bind to the GPR43 receptor on placental endothelial cells, enhancing the tubule-forming capacity of trophoblasts, maintaining normal blood perfusion in the placental labyrinth, and facilitating the physiological remodeling of spiral arteries (Grade 3, *in vitro* trophoblast/placental endothelial cell studies) (42, 43).

Pathological Imbalance-Induced Damage Hypothesis: SCFA deficiency leads to a sparse placental vascular network, reduced placental weight, and insufficient fetal oxygen supply, potentially causing fetal growth restriction (FGR) (Level 1, germ-free animal model) (44). SCFA deficiency and downregulation of receptor expression resulting from gut microbiota dysbiosis during pregnancy are major contributors to abnormal placental vascular development (45).

#### 4.1.4 Potential hypotheses regarding epigenetic *in utero* programming

Physiological Regulation (Normal Pregnancy): Physiological doses of butyrate modulate fetal chromatin via HDACs, moderately regulating immune, neural, and metabolic genes, thereby establishing a baseline for healthy fetal development.

Pathological Imbalance-Induced Damage Hypothesis: Both SCFA deficiency and abnormally high concentrations of butyrate can disrupt epigenetic modifications, increasing the risk of long-term metabolic disorders and autoimmune diseases in offspring (Level 3: *in vitro* cellular/placental organoid evidence; Level 4: epigenetic inferences from animal studies, animal observations only, no human validation) (39, 46).

### 4.2 Tryptophan metabolites (kynurenine, indole-3-propionic acid)

Physiological Regulation (Normal Pregnancy): During pregnancy, Lactobacillus and Bacteroides break down intestinal tryptophan to produce indole derivatives, which indirectly regulate the host’s indoleamine 2,3 -dioxygenase (IDO) activity, thereby influencing production in kynurenine activating the aryl hydrocarbon receptor (AhR) pathway (47). The AhR pathway is a key regulatory pathway for maternal-fetal immune tolerance: decidual dendritic cells induce Treg generation via AhR signaling, upregulate IDO, and locally deplete tryptophan to inhibit excessive proliferation of allogeneic T cells (38); indole-3-propionic acid can strengthen the intestinal mucosal barrier and reduce LPS leakage into the bloodstream (48).

Pathological Imbalance-Induced Damage Hypothesis: Animal and population-based association studies suggest that low tryptophan intake during pregnancy and a deficiency in tryptophan-metabolizing gut microbiota can silence the AhR pathway, leading to an abnormal accumulation of cytotoxic T cells at the maternal-fetal interface and significantly increasing the risk of early embryonic loss and recurrent spontaneous abortion (RSA) (Level 1, FMT animal studies, animal causal validation; Level 2: human pregnancy cohorts, population-based association evidence) (49). The microbiota–tryptophan–AhR axis has been identified as a potential pathway involved in regulating immune homeostasis in female fertility (50, 51).

### 4.3 Bile acids and their derivatives (potential dose-dependent bidirectional effects)

Physiological Regulation (Normal Pregnancy): Primary bile acids produced by the liver are deoxygenated by the gut microbiota to form secondary bile acids. During pregnancy, there is a dose-dependent bidirectional effect: Low concentrations of ursodeoxycholic acid (UDCA) can protect trophoblast viability, alleviate oxidative stress damage to the placenta, maintain endoplasmic reticulum homeostasis, and potentially ensure normal EVT invasion (Level 2: evidence from ICP patient cohorts and population-based studies; Level 3: *in vitro* trophoblast experiments and *in vitro* hypothetical evidence) (52).

Pathological Imbalance-Induced Damage Hypothesis: Excessive accumulation of toxic secondary bile acids, such as high concentrations of deoxycholic acid and chenodeoxycholic acid, can mediate placental cell damage through endoplasmic reticulum stress, thereby impairing the normal invasive function of extra-villus trophoblasts (EVTs).Patients with intrahepatic cholestasis of pregnancy (ICP) commonly exhibit disruptions in gut microbiota composition. The accumulation of toxic secondary bile acids disrupts placental transport capacity via the gut-placenta axis, thereby increasing the risk of fetal intrauterine hypoxia, spontaneous preterm birth, and fetal growth restriction (FGR) (Level 2, evidence from ICP case cohorts and population-based studies; Level 3: *in vitro* cellular experiments and *in vitro* hypothesis-based evidence) (53, 54). Conclusions regarding the bidirectional regulation of bile acids are based solely on *in vitro* cellular studies and patient microbiome association analyses; no randomized human intervention trials have confirmed a direct causal relationship between bile acid concentrations and pregnancy outcomes (55).

### 4.4 Metabolites of harmful bacterial communities: potential damaging mediators that disrupt immune tolerance

Under normal physiological conditions, the intestinal barrier is intact, and only trace amounts of LPS, TMAO, and branched-chain amino acids (BCAAs) enter the bloodstream, which does not activate systemic inflammatory pathways; it is only when dysbiosis and increased intestinal permeability occur that large accumulations of these substances lead to disease.

#### 4.4.1 Lipopolysaccharide

Under physiological conditions, the intact intestinal barrier prevents the entry of Gram-negative bacterial cell wall components, and circulating LPS levels remain below the detection limit; under pathological conditions, when intestinal permeability increases, large amounts of LPS are translocated into the bloodstream, continuously activating the TLR4/NF-κB pathway, potentially inducing chronic inflammation of the placenta, which may mediate a common upstream cause of PE, RSA, and preterm birth (Level 1, LPS animal models, animal causality validation; Level 2: evidence from pregnancy case cohorts and population-based associations) (5, 30, 56).

#### 4.4.2 Trimethylamine Oxide

Under physiological conditions, with a normal low-fat diet and a balanced gut microbiota, TMAO synthesis is extremely low and does not interfere with vasodilation; under pathological conditions, excessive red meat consumption and gut microbiota imbalance drive TMAO accumulation, which may impair vasodilation and potentially hinder spiral artery remodeling, thereby increasing the risk of gestational hypertension (57). Metabolic disorders potentially driven by an imbalance in the gut microbiota are a key pathophysiological mechanism underlying preeclampsia, and the microbiota-TMAO metabolic axis is increasingly recognized as a potential biomarker and intervention target for gestational hypertension (Level 2; preeclampsia metabolomics cohort, population-based association evidence only) (58). Only statistical associations have been observed in population studies; to date, no randomized controlled clinical trials in humans have verified a causal relationship between elevated TMAO levels and preeclampsia, and therefore this cannot be used to establish a definitive pathogenic mechanism.

#### 4.4.3 Branched-chain amino acids

Under physiological conditions, with a normal, balanced diet and moderate protein fermentation in a stable gut microbiota, BCAAs maintain physiological concentrations and do not interfere with insulin signaling;Under pathological conditions, dysbiosis leads to abnormal protein fermentation; elevated BCAA levels may induce maternal insulin resistance and potentially suppress the expression of glucose transporters (GLUT), thereby contributing to the development of GDM and macrosomia (Level 2 evidence from GDM multi-omics cohorts and population-based association studies) (59–61). Correlations have been observed only in population-based multi-omics studies; no animal FMT or human intervention studies have established a causal effect of BCAAs directly leading to GDM (60, 61).

## 5 A pathological hypothesis: how dysbiosis of the gut-placenta axis mediates various adverse pregnancy outcomes

**Figure 2:**
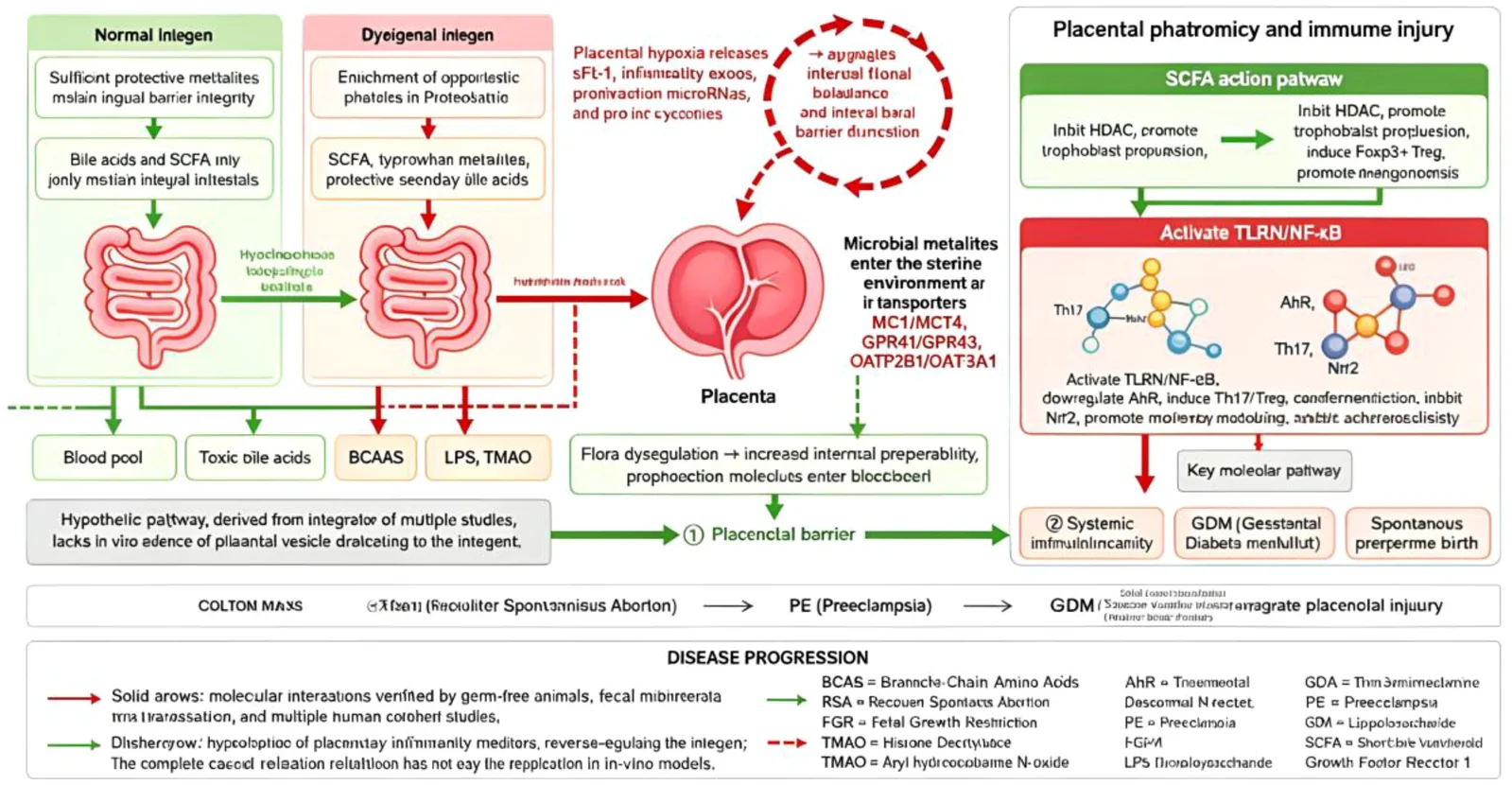
Schematic representation of the bidirectional regulatory loop within the gut–placenta axis.

All adverse pregnancy outcomes are mediated by a combination of genetic, immunological, vascular, and metabolic factors; metabolic dysregulation of the gut-placental axis microbiota is merely a potential regulatory pathway and is not the primary or sole causative factor. We should avoid oversimplifying the pathogenesis by attributing it to a single cause. A stable gut microbiota exerts a protective effect on pregnancy maintenance. Based on evidence from population-based cohorts, germ-free animals, and FMT interventions, this study systematically maps the complete pathogenic pathways of gut-placental axis dysregulation across various diseases and distinguishes the microbiota profiles and target sites of damage associated with different pregnancy complications as shown in **Figure 2**.

### 5.1 Recurrent miscarriage

Recurrent spontaneous abortion (RSA) refers to two or more consecutive clinical pregnancy losses and is a common pregnancy complication among women of childbearing age.Patients exhibit reduced abundance of SCFA-producing microbiota in their feces, excessive enrichment of *Pseudomonas* species and *E. coli* within the *Proteobacteria* phylum, and increased intestinal permeability (62), which triggers a cascade of damage via the gut-placental axis: depletion of protective metabolites such as butyrate and indole derivatives (63), which inhibits Treg differentiation and potentially drives excessive IFN-γ secretion by Th1 cells and abnormal activation of uNK cells, resulting in insufficient trophoblast invasion, implantation failure, and defects in placental vascular development (64, 65); At the same time, bacterial translocation releases LPS, which activates the TLR4 pathway, inducing chronic inflammation at the maternal-fetal interface and promoting trophoblast apoptosis and embryonic arrest (65). Animal studies on fecal microbiota transplantation (FMT) can replicate the high-miscarriage phenotype; exogenous supplementation with short-chain fatty acids (SCFAs) can reshape maternal-fetal immune tolerance and reduce the rate of embryonic loss, providing experimental evidence for clinical intervention (Level 1: animal intervention studies on FMT and animal causal validation; Level 2: RSA case cohorts and population-based association evidence) (64).

### 5.2 Preeclampsia

The most extensively studied complication involving the intestine-placenta axis involves a bidirectional pathological feedback loop (5).Microbiota characteristics are manifested by reduced microbial α-diversity, depletion of Akkermansia and Bacteroides, enrichment of pro-inflammatory bacteria such as Veillonella and Fusobacterium, decreased fecal SCFAs, and abnormally elevated levels of LPS and TMAO in circulating plasma (66, 67). The pathogenesis of this condition can be divided into two levels: ① Deficiency of protective metabolites: Insufficient supply of SCFAs (particularly reduced butyrate) has been confirmed in clinical cohorts of PE (68); GDM studies have also shown that reduced SCFAs are associated with elevated HDAC expression in the placenta (19), suggesting that SCFA deficiency may inhibit HDAC-mediated epigenetic regulation, thereby weakening the Nrf2 antioxidant response (69) and vasodilatory function (70), and inducing oxidative stress damage in the decidua and placental endothelium, as well as superficialization of spiral artery remodeling (71).It should be noted that changes in Nrf2 differ between PE subtypes with and without FGR, and evidence of vasodilation and remodeling is primarily derived from non-pregnancy models; placenta-specific validation is still needed;② Accumulation of toxic molecules: LPS potentially drives systemic low-grade inflammation; a damaged, hypoxic placenta releases large amounts of sFlt-1 and anti-angiogenic exosomes, which are reabsorbed into the intestine, further disrupting the intestinal barrier and microbiota homeostasis, thereby creating a cascading, amplifying vicious cycle (Level 1: animal models validating a unidirectional damage pathway; Level 2: nested case-control cohorts and population-based association evidence; Level 4: integrated inferential hypothesis) (5).

The complete feedback loop is based solely on integrated inferential hypotheses, with no direct evidence from *in vivo* tracing. A dual pathophysiological effect jointly mediates widespread maternal vasospasm, hypertension, and proteinuria, accompanied by placental hypoperfusion, leading to fetal growth restriction (FGR) and iatrogenic preterm birth; in severe cases, this may progress to multi-organ dysfunction in both mother and infant (72, 73). A nested case-control cohort study suggests that fecal SCFA levels in the second trimester of pregnancy may serve as a candidate noninvasive biomarker for the early prediction of PE; however, evidence of screening efficacy is limited to population-level associations (73) and requires validation through large-scale RCTs, and long-term safety remains unclear.

### 5.3 Gestational diabetes

Obesity and a high-sugar diet can induce gut microbiota dysbiosis during pregnancy, characterized by a significantly elevated Firmicutes/Bacteroidetes (F/B) ratio, hyperactive branched-chain amino acid (BCAA) and TMAO anabolic pathways, and insufficient short-chain fatty acid (SCFA) production. Microbiota metabolic dysregulation occurs through two potential pathways: ① Peripheral insulin resistance pathway: Deficient SCFA secretion directly reduces the release of intestinal glucagon-like peptide-1 (GLP-1), leading to maternal peripheral insulin resistance, glucose metabolism disorders, and persistent hyperglycemia (74); ② Placental dysfunction pathway: Maternal hyperglycemia, in combination with toxic metabolites such as BCAAs and TMAO, collectively inhibits placental GLUT expression, leading to fetal hyperinsulinemia and macrosomia, while simultaneously increasing the offspring’s long-term risk of metabolic diseases such as obesity and type 2 diabetes (75). Randomized controlled dietary interventions have demonstrated that a high-fiber diet reshapes the butyrate-producing microbiota, increases circulating SCFAs, improves insulin sensitivity, and reduces the risk of GDM (Level 1: randomized controlled dietary intervention trials, causal evidence from human studies; Level 2: association studies in GDM cohorts) (76).

### 5.4 Fetal growth restriction

The target of damage mediated by the gut-placental axis in FGR is impaired placental vascular development; the pathogenesis of this functional dysfunction (animal/associative evidence): A deficiency of butyrate-producing bacteria in the maternal gut leads to insufficient levels of circulating short-chain fatty acids (SCFAs), which downregulate the expression of monocarboxylic acid transporters (MCTs) in the placenta and inhibit the secretion of vascular endothelial growth factor (VEGF) by trophoblasts. This results in a decrease in placental villous vascular density and impaired transplacental exchange of oxygen and nutrients. Meanwhile, LPS-mediated chronic placental inflammation can induce microthrombosis in the villi, further exacerbating fetal hypoxia and potentially increasing the risk of small for gestational age (SGA).Small-for-gestational-age (SGA) infants are defined as those with a fetal weight below the 10th percentile for their gestational age. Fetal growth restriction (FGR) is characterized by persistent intrauterine growth retardation caused by maternal or placental pathological factors; it may be associated with SGA, but some cases of SGA represent a physiological constitutional variant without placental dysfunction. Imbalances in the gut-placental axis contribute only to the pathogenesis of pathological FGR and do not affect physiologically small-for-gestational-age fetuses; Animal studies have shown that germ-free pregnant mice naturally exhibit phenotypes of placental hypoplasia and small fetal size, whereas exogenous SCFA intervention can effectively restore placental volume and vascular networks, thereby providing reverse evidence of the potential causal role of SCFA deficiency in placental vascular abnormalities and FGR (Level 1, germ-free animal model, animal evidence of causality) (77).

### 5.5 Spontaneous preterm birth

Disruption of the gut microbiota leads to increased intestinal permeability and triggers systemic low-grade chronic inflammation, which is a contributing factor to spontaneous preterm birth. Increased intestinal barrier permeability results in the intestinal translocation of LPS into the peripheral circulation, where circulating LPS acts in concert with pro-inflammatory factors such as tumor necrosis factor-α (TNF-α) and IL-6 via the gut -placental axis to act on the maternal-fetal interface: on the one hand, it stimulates trophoblasts to release large amounts of matrix metalloproteinases, which degrade the collagen fiber structure of the amniotic membranes, leading to preterm rupture of membranes; on the other hand, it continuously activates signaling pathways for uterine muscle contractions, inducing pathological uterine contractions, which ultimately result in preterm delivery before full term (78–81); Inappropriate use of broad-spectrum antibiotics during pregnancy directly disrupts the structure of the gut microbiome, compromises the integrity of the intestinal barrier, depletes beneficial bacteria that produce short-chain fatty acids (SCFAs) on a large scale, reduces the body’s reserves of protective SCFAs, weakens the anti-inflammatory homeostasis of the gut-placental axis, and significantly increases the risk of preterm birth. This indirectly confirms that the gut -placental axis homeostasis is crucial for maintaining a full-term pregnancy (Level 1: LPS-induced preterm birth animal models and animal causal validation; Level 2: population-based cohorts of women exposed to antibiotics during pregnancy, population-based associative evidence) (82–85).

## 6 Controversies and limitations in current research on the gut-placenta axis

In the field of the gut-placental axis, from the introduction of the concept in 2018 through 2026, although a body of evidence has been accumulated from germ-free animal models, fecal microbiota transplantation, human cohort studies, and *in vitro* organoids, significant academic disagreements, gaps in evidence, and inherent research limitations persist regarding core mechanisms, human translation, and clinical applications. These issues require validation through large-scale randomized controlled trials (RCTs), and long-term safety remains unclear; therefore, routine clinical use is not currently recommended.

### 6.1 Controversy surrounding microbial colonization of the placenta

Currently, there are two opposing hypotheses in the academic community regarding the intrauterine microbiome of the placenta: the placental sterility hypothesis and the trace placental microbiota hypothesis.

#### 6.1.1 The placental aseptic hypothesis (the prevailing view in basic research)

Under physiological conditions, no live bacteria can cross the intestinal-placental barrier to colonize placental tissue; the trace bacterial nucleic acid signals detected by high-throughput sequencing of the placenta essentially originate from ascending contamination of the genital tract during childbirth, as well as technical background contamination from experimental reagents and sequencing procedures, and therefore have no true biological significance (Level 2, evidence from sequencing cohorts of human placental specimens and population-based studies) (86, 87);

#### 6.1.2 The hypothesis of trace placental microorganisms (a pathology-oriented research perspective)

Under pathological conditions of severely increased intestinal permeability, bacterial fragments and extracellular vesicles of intestinal origin can cross the compromised barrier and accumulate locally in the placenta via the bloodstream; without the need for live bacterial colonization, they can activate local signaling pathways and amplify low-grade inflammatory responses at the maternal-fetal interface (Grade 3, *in vitro* experiments incubating placental cells with bacterial fragments; *in vitro* hypothetical evidence) (88).

Neither of the two hypotheses has been confirmed by human clinical trials using standardized, aseptic sampling methods; while the current evidence for one mechanism is relatively robust, it remains inconclusive whether trace microbial colonization occurs in the placenta.

#### 6.1.3 The roots of disagreement and areas of consensus between the two opposing hypotheses

The crux of the long-standing disagreement between these two viewpoints lies in inherent contamination risks in the human placenta specimen collection process: During placental separation in labor, the placenta inevitably comes into contact with the microbial flora of the cervix and the vaginal tract. Without standardized aseptic and contamination-prevention protocols throughout the entire process—including surgery, transport, lysis, and sequencing—trace microbial signals cannot be distinguished as originating from the placenta itself or from external contamination;

Limitations of microbial detection technologies for low-biomass samples: The microbial load in placental tissue is significantly lower than that in the gut and reproductive tract; 16S rRNA sequencing lacks sufficient sensitivity; background bacteria in the reagents can easily interfere with result interpretation; and there is currently no standardized calibration algorithm in the industry;

Missing stratification in study populations: Most studies have failed to distinguish between stratified groups such as normal pregnancy, preeclampsia (PE), recurrent spontaneous abortion (RSA), and severe obesity with increased intestinal permeability; they have not separately compared differences in physiological versus pathological placental microbial signatures, resulting in excessive confounding variables. This discrepancy is unlikely to be fully resolved in the short term (17, 87).

Regardless of the positions taken by the two major hypotheses, the academic community has reached a consensus: gut-placental signaling mediated by soluble microbial metabolites may be involved in regulating maternal-fetal immune tolerance and placental development through potential pathways, and this mechanism is not affected by the presence or absence of trace microbial components in the placenta; The transplacental transport of short-chain fatty acids (SCFAs), lipopolysaccharides (LPS), trimethylamine oxide (TMAO), and tryptophan derivatives, as well as their potential role in regulating the immune system, has been repeatedly validated by multiple independent studies. This represents a consensus conclusion supported by relatively robust evidence in the field; however, specific regulatory doses, species differences, and effects in humans remain to be further clarified (89).

### 6.2 Heterogeneity in the study population makes it difficult to reach consistent conclusions

#### 6.2.1 Uncontrollable, multi-level confounding variables from the study population interfere with the baseline microbial community

Differences in dietary patterns: There are significant differences between Eastern and Western diets in the intake of dietary fiber, red meat, and fermented foods, which directly alter the baseline composition of SCFA-producing microbiota and TMAO-synthesizing microbiota;

Pre-pregnancy baseline status: pre-pregnancy BMI, obesity, polycystic ovary syndrome, chronic gastrointestinal diseases, and a history of pre-pregnancy antibiotic use reshape the long-term homeostasis of the maternal gut microbiota;

Stages of Pregnancy: Hormonal fluctuations throughout early, middle, and late pregnancy continuously alter the composition of the microbiota; however, because different studies use inconsistent gestational weeks for sampling, the results cannot be compared across studies;

Exposure to external interventions: Antibiotics during pregnancy, probiotics, iron and calcium supplements, psychological stress, and geographic, racial, and genetic background may play a role in regulating the gut microbiota (Level 2, evidence from multicenter pregnancy cohorts and population-based studies) (90, 91).

#### 6.2.2 Limited multi-omics testing strategies and gaps in the chain of evidence

Currently, the vast majority of clinical studies only perform metagenomic sequencing of fecal microbiota, which presents three types of evidence gaps:

Since the study only examined fecal microbiota and lacked combined validation using maternal plasma metabolomics, placental tissue transcriptomics, and immunophenotyping of decidual cells, it can only demonstrate that “microbiota abundance is associated with adverse pregnancy outcomes”; it cannot distinguish whether microbiota dysbiosis induces complications or whether complications lead to secondary microbiota dysbiosis; Lack of dynamic longitudinal sampling: Sampling is typically limited to single-point cross-sectional measurements, making it impossible to track the dynamic evolution of the microbiome from the preconception period through early, mid, and late pregnancy, and resulting in a lack of evidence for temporal causality; Tests for placental functional markers (sFlt-1, placental vascular density, and trophoblast invasive capacity) are rarely performed, making it impossible to establish a complete chain of association between “microbiota—metabolites—placental damage—pregnancy outcomes” (92, 93).

#### 6.2.3 There is a natural species barrier to translating conclusions from animal experiments to humans

Germinally sterile animals and rodent models of fecal microbiota transplantation can establish a causal relationship between microbial dysbiosis and adverse pregnancy outcomes in animal systems (Level 1, animal causal evidence; applicable only to rodent systems), but this cannot be directly extrapolated to humans: the core gut microbiota compositions of mice and humans differ significantly, and the dominant microbial communities in mice cannot replicate the structure of human microbial communities involved in butyrate and tryptophan metabolism; The structure of the placenta, the pattern of spiral artery remodeling, and the maternal-fetal immune regulatory pathways (Treg and uNK cell subsets) in rodents differ fundamentally from those in humans; Hormonal fluctuations during pregnancy in mice, dietary metabolic patterns, and the expression profile of placental transport proteins do not correspond to those in humans; existing causal evidence from animal studies can only suggest potential mechanisms but cannot serve as conclusive evidence for human pathogenesis (Level 4: cross-species extrapolation hypothesis, not verified in humans) (94).

### 6.3 Evidence on the dosage and safety of perinatal microbiome interventions is unclear

Dietary fiber, probiotics/prebiotics, and exogenous SCFA supplementation are currently the most common intervention strategies for gut microbiota dysbiosis during pregnancy; however, there are still significant gaps in the clinical evidence in this field: There is a relative lack of large-scale, randomized controlled trials involving long-term follow-up of mothers and infants; consequently, no uniform standards have been established regarding the gestational week at which intervention should begin, the effective dosage of supplements, or the formulation of probiotic strains (Level 2; evidence from small, short-term intervention cohorts and weak population-level associations) (95). In terms of safety, high doses of SCFAs and tryptophan-derived microbial metabolites during pregnancy can mediate fetal epigenetic modifications; however, the potential adverse developmental outcomes resulting from *in utero* exposure to these substances have not yet been fully evaluated in human studies. Due to insufficient evidence and uncertainty regarding long-term safety, targeted interventions targeting the perinatal gut microbiome have not yet been incorporated into standard clinical guidelines for obstetrics and gynecology (96). All microbiome interventions show only a potential protective trend; their safety and efficacy have not been confirmed by high-level human trials, and they should not be used as routine obstetric prevention or treatment measures. Their long-term safety and standardized efficacy remain to be verified by large-scale randomized controlled trials (RCTs).

### 6.4 There are significant gaps in our understanding of the molecular mechanisms underlying bidirectional regulation of the gut-placenta axis

The specific molecular pathways through which placental exosomes and inflammatory factors reciprocally regulate the gut microbiota remain unclear (97). The complex synergistic and antagonistic effects among various microbial metabolites, as well as the cross-regulatory network formed by these metabolites and pregnancy hormones (estrogen and progesterone), have not yet been fully elucidated (Level 4: Incomplete integration of inferences; all are hypotheses awaiting verification) (98). There is no direct *in vivo* tracing or organoid-based validation of the complete bidirectional feedback molecular signaling pathway; these remain purely theoretical speculations, and no definitive conclusions regarding the mechanism have been reached.

## 7 Future research directions and clinical applications

### 7.1 Establishing a multi-omics integrated early Nnon-invasive early warning system

By integrating multi-omics data—including fecal metagenomics, plasma metabolomics, and peripheral immune cell profiling—from the preconception and early pregnancy periods, and leveraging machine learning to construct risk prediction models, we aim to identify candidate non-invasive circulating biomarkers such as SCFAs, indole derivatives, and TMAO, with the goal of exploring a model validation pathway for risk stratification of RSA, PE, and GDM 3–6 months in advance; This predictive framework is currently only a theoretical modeling concept and does not imply that existing biomarkers can be directly used for clinical screening; it requires validation through large-scale prospective cohort studies and randomized controlled trials (RCTs). It may provide a basis for future research on proactive, personalized interventions for high-risk populations (Level 4, integrated inference hypothesis) (99, 100).

### 7.2 A future research framework for stratified interventions targeting the perinatal microbiome

Based on evidence from existing animal studies, *in vitro* models, and small-scale population intervention studies, interventions targeting the gut microbiome during pregnancy can be tentatively categorized into a three-tiered research framework, rather than established clinical prevention and treatment protocols: The first line of research focuses on the general population of pregnant women. A personalized diet rich in highly soluble dietary fiber may endogenously stimulate SCFA synthesis; with relatively robust evidence regarding its safety, it can serve as a potential strategy for optimizing the prenatal diet, and its value for routine prevention should be further evaluated in large-scale RCTs (Grade 2, small-scale dietary fiber RCTs, population-based evidence) (101); The second research direction focuses on populations at high risk for recurrent spontaneous abortion (RSA) and preeclampsia (PE). It explores probiotic and postbiotic intervention strategies, with candidate strains including Akkermansia muciniphila, Clostridium butyricum, and Lactobacillus plantarum—among which C. butyricum is the primary butyrate-producing bacterium, while A. muciniphila and L. plantarum are characterized by propionate and lactic acid metabolism, respectively—or directly supplementing with purified SCFAs or indolepropionic acid postbiotics to avoid the risk of unstable live-bacteria colonization; their efficacy and safety remain to be validated by prospective clinical trials (Level 2, small obstetric intervention cohorts, weak evidence) (102); Level 3 research focuses on salvage studies for refractory cases and may only be conducted as clinical trials in populations with refractory recurrent miscarriage or severe obesity complicated by pregnancy-related complications, where conventional microbiome interventions have proven ineffective. Strict control must be exercised over the timing of transplantation, donor screening, and aseptic techniques, and clinical trial inclusion and exclusion criteria must be strictly defined (Level 1: FMT effective in animals; only case reports in humans; no large-scale RCTs) (103). The three-tiered approach described above serves only as a framework for future stratified research. Currently, there are no standardized guidelines regarding dosage, gestational age at intervention, or bacterial strain combinations. Fecal microbiota transplantation is applicable only to basic research and clinical trials involving refractory cases; no clinical indications have been established, and it has not yet been incorporated into routine obstetric clinical practice.

### 7.3 Research directions for in-depth study of fundamental mechanisms

Future research could refine the research framework for the gut-placental axis mechanism at three levels: establishing standardized operating procedures for aseptic placental sampling; clarifying the damaging effects of trace microbial fragments on placental tissue under conditions of severe increased intestinal permeability; and avoiding experimental bias caused by contamination during sample collection (104); Using an *in vitro* model of human placental organoids, investigate the regulatory effects of individual microbial metabolites on trophoblasts and decidual immune cells in isolation, thereby eliminating complex physiological confounding factors *in vivo* (Level 3, *in vitro* hypothetical evidence) (105); Elucidate the complete molecular pathway by which placental exosomes reciprocally regulate the maternal gut microbiota, complete the closed-loop of bidirectional signaling in the gut-placenta axis, and refine the overall theoretical model of this axis (Level 4, hypothesis requiring validation).

### 7.4 Expanding the long-term follow-up cohort covering the entire lifecycle of mothers and infants

Existing studies have only examined short-term adverse pregnancy outcomes; there remains a lack of long-term follow-up data from large-scale birth cohorts to support the long-term effects of SCFAs in inducing childhood obesity, autoimmune diseases, and neurodevelopmental abnormalities in offspring by regulating fetal epigenetic modifications (Level 4, hypothetical inference regarding long-term effects);Future research should be grounded in the theory of the full life cycle of mothers and infants to clarify the comprehensive clinical value of the gut-placental axis in the prevention and control of long-term chronic diseases in offspring (106).

## 8 Conclusion

The gut-placental axis serves as a communication bridge linking the microbiome of the distal maternal intestine with the immunological balance at the maternal-fetal interface and placental development. Various gut microbial metabolites act as signaling molecules; in animal and *in vitro* models, they have been shown to participate in regulating physiological processes such as the Treg/Th17 balance, the polarization of decidual macrophages, and the remodeling of placental spiral arteries. However, causal evidence in humans remains insufficient for most of these findings. When various factors during pregnancy disrupt the balance of the gut microbiota, potentially protective metabolites such as SCFAs and tryptophan-indole derivatives are depleted, while pro-inflammatory and toxic molecules such as LPS, TMAO, and toxic secondary bile acids accumulate. Through the intestinal -placental axis, these molecules interfere with placental function and disrupt maternal-fetal immune tolerance through multiple pathways. Existing evidence from animal studies, *in vitro* experiments, and population-based associations suggests that microbial metabolic dysregulation may contribute to the development of these complications; however, a complete causal chain still requires confirmation through large-scale human intervention trials.

There are still multiple limitations in this field: there remains a long-standing academic debate regarding whether trace colonization of the placental microbiome exists; confounding variables such as diet, body weight, and gestational age make it difficult to reach consistent conclusions in cohort studies; the placental-to-gut feedback pathway remains only a unifying hypothesis, with the complete molecular mechanism yet to be elucidated and no direct evidence from *in vivo* experiments; and microbiome interventions such as dietary fiber and probiotics lack standardized dosing regimens, and there is insufficient long-term human evidence regarding their safety for both mother and infant, making it impossible to incorporate them into routine obstetric clinical guidelines at this time.

The gut-placenta axis theory broadens the scope of research into the pathogenesis of pregnancy complications, moving beyond the previous narrow focus on local uterine immunity. In the future, by leveraging standardized protocols for aseptic placental sampling, *in vitro* models of human placental organoids, and large-scale, multi-omics integrated pregnancy cohorts, we can further elucidate the intricate regulatory networks of microbial metabolites and establish a preconception - early pregnancy, explore safe and standardized perinatal microbiome interventions, and gradually shift the management of adverse pregnancy outcomes from passive postpartum treatment to proactive prevention and control during the preconception and early pregnancy periods, ultimately improving long-term health outcomes for both mothers and their offspring. All candidate biomarkers and stratified intervention strategies are intended solely as directions for future research; they have not yet been developed into mature clinical protocols, require validation through large-scale RCTs, and their long-term safety remains unclear. Therefore, their routine clinical use is not currently recommended.

## APPENDIX

**Table:** Appendix 1 Summary table of metabolite sources, transport receptors, immune targets, levels of evidence, and associated adverse pregnancy outcomes for the core gut microbiota.

Metabolite categories	Specific metabolites	Source (core microbial community/formation pathway)	Transporter/transporter protein	Immune targets/molecular mechanisms	Level of evidence	Associated with adverse pregnancy outcomes
Short-chain fatty acids (SCFAs)	Acetic acid, propionic acid, butyric acid (accounting for more than 90% of the total)	Bacteroides, Bifidobacterium, Clostridium butyricum, and other bacteria ferment dietary fiber to produce	MCT1/MCT4 (transmembrane transporters); GPR41/GPR43 (specific membrane receptors, highly expressed in trophoblasts and decidual macrophages)	① HDAC inhibition → upregulation of Foxp3 → induction of Treg homing to the decidua, where they secrete IL-10/TGF-β to suppress Th17; ② HDAC inhibition downregulates NF-κB and upregulates Nrf2 for antioxidant effects → M2 polarization of macrophages; ③ Binding to GPR43 → enhances trophoblast tubule formation → promotes placental vascular development; ④ Butyrate-mediated HDAC modification of fetal chromatin → epigenetic programming *in utero*	Level 1: Germ-free animals, isotope tracing (labeled butyric acid detectable in fetal mouse liver, brain, and placenta), FMT animal studies, L-NAME-induced PE rat model with SCFA supplementation; Level 2: Cohort of healthy pregnant women, PE case-control study (n=20 per group), RSA cohort; Level 3: *In vitro* cell/placental organoids; Level 4: Animal-based extrapolation	RSA, PE, GDM, FGR, Spontaneous Preterm Birth
Tryptophan Metabolites	kynurenine, indole-3-propionic acid (IPA), indole-3-methanol	Lactobacillus and Bacteroides break down tryptophan in the gut to produce indoles/kynurenine	OATP2B1/OATP3A1 (transplacental transport); AhR (aromatic hydrocarbon receptor, intracellular receptor)	① Activation of the AhR pathway → Induction of Treg generation by decidual dendritic cells; ② Upregulation of IDO → Local depletion of tryptophan → Inhibition of allogeneic T-cell overproliferation; ③ Indole-3-propionic acid strengthens the intestinal mucosal barrier → Reduces LPS leakage into the bloodstream; ④ The microbiota–tryptophan–AhR axis regulates immune homeostasis in female fertility	Level 1: FMT animal studies (colonization of germ-free mice with Lactococcus lactis producing tryptophan metabolites restores immune balance and reduces fetal absorption), oral indole-3-methanol supplementation intervention; Level 2: Human pregnancy cohort, RSA decidual tissue validation (MDSCs/RORγt^+^ Tregs/tryptophan metabolite dysregulation)	RSA, early embryonic loss, recurrent miscarriage
Bile acids and their derivatives	Ursodeoxycholic acid (UDCA, protective); deoxycholic acid, chenodeoxycholic acid (toxic secondary bile acids)	The liver synthesizes primary bile acids → which are deoxygenated and modified by the gut microbiota to form secondary bile acids; a dose-dependent bidirectional effect is observed during pregnancy.	OATP2B1/OATP3A1 (highly expressed in the placenta; mediates the transplacental transport of bile acids)	① Low-concentration UDCA: protects and nourishes cellular vitality, alleviates oxidative stress-induced damage to the placenta, maintains endoplasmic reticulum homeostasis, and ensures normal EVT invasion (protective effects); ② High-concentration toxic secondary bile acids: induce endoplasmic reticulum stress, which mediates placental cell damage, impairs EVT invasion, and disrupts placental substance transport capacity	Level 2: ICP patient cohorts (gut microbiota dysbiosis, accumulation of toxic secondary bile acids), population-based evidence; Level 3: *in vitro* trophoblast experiments, *in vitro* hypothetical evidence; no randomized human intervention trials have confirmed a direct causal relationship between bile acid concentrations and pregnancy outcomes	ICP (Intrahepatic Cholestasis of Pregnancy), Fetal Intrauterine Hypoxia, Spontaneous Preterm Birth, FGR
Lipopolysaccharide (LPS)	Lipopolysaccharide Components of the Cell Walls of Gram-Negative Bacteria	Excessive proliferation of Gram-negative opportunistic pathogens in the order Proteobacteria (such as Proteus mirabilis and Escherichia coli) leads to their release; when intestinal permeability increases, large numbers of these bacteria translocate into the bloodstream.	Passive diffusion into the bloodstream following disruption of the intestinal barrier; TLR4 (Toll-like receptor 4, a membrane receptor) → NF-κB inflammatory pathway	Sustained activation of the TLR4/NF-κB inflammatory pathway → induces chronic inflammation in the placenta → promotes trophoblast apoptosis and embryonic arrest; mediates a common upstream cause of PE, RSA, and preterm birth; synergizes with TNF-α/IL-6 → stimulates amniotic trophoblasts to release matrix metalloproteinases → degrades amniotic membrane collagen → premature rupture of membranes; activates uterine myometrial contraction signals → pathological uterine contractions	Level 1: LPS animal models (LPS-induced preterm birth in mice, PE in rats), animal studies to establish causality; Level 2: Cohorts of pregnancy cases, elevated plasma LPS levels in PE patients, cohorts of individuals exposed to antibiotics during pregnancy	PE, RSA, preterm labor, FGR, premature rupture of membranes
Trimethylamine Oxide (TMAO)	Trimethylamine N-oxide	Excessive red meat intake + gut microbiota imbalance → Gut microbiota metabolize choline and L-carnitine to produce TMA → TMA is oxidized to TMAO by liver flavin monooxygenase	Distribution occurs primarily via passive diffusion; placental-specific transporters remain to be identified.	Impaired vascular vasodilation → Potential hindrance to spiral artery remodeling → Increased risk of gestational hypertension; the gut microbiota–TMAO metabolic axis is considered a potential biomarker and intervention target for gestational hypertension	Level 2: Preeclampsia Metomics Cohort (abnormally elevated plasma TMAO levels in patients with PE); evidence is limited to population-based associations; to date, no randomized controlled clinical trials in humans have established a causal relationship between elevated TMAO levels and PE.	PE, Hypertensive Disorders of Pregnancy
Branched-Chain Amino Acids (BCAAs)	Leucine, Isoleucine, Valine	Gut microbiota dysbiosis → Abnormal protein fermentation → Elevated BCAA levels; physiological concentrations are maintained with a normal, balanced diet and stable gut microbiota.	Distribution occurs primarily via passive diffusion; placental-specific transporters remain to be identified.	Induction of maternal insulin resistance → potential suppression of glucose transporter (GLUT) expression → impaired glucose transport in the placenta → fetal hyperinsulinemia and macrosomia; SCFA deficiency reduces GLP-1 release → peripheral insulin resistance and impaired glucose metabolism	Level 2: GDM multi-omics cohorts (elevated F/B ratio, hyperactive BCAA anabolic pathways), population-based association evidence; no animal FMT or human intervention studies have established a direct causal relationship between BCAAs and GDM	GDM, macrosomia, and the risk of obesity and type 2 diabetes in offspring later in life
